# Lactate promotes specific differentiation in bovine granulosa cells depending on lactate uptake thus mimicking an early post-LH stage

**DOI:** 10.1186/s12958-018-0332-3

**Published:** 2018-02-20

**Authors:** Anja Baufeld, Jens Vanselow

**Affiliations:** Institute of Reproductive Biology, Leibniz Institute for Farm Animal Biology (FBN), Wilhelm-Stahl-Allee 2, 18196 Dummerstorf, Germany

**Keywords:** Bovine, Granulosa cells, L-lactate, Expression

## Abstract

**Background:**

The LH-induced folliculo-luteal transformation is connected with alterations of the gene expression profile in cells of the granulosa layer. It has been described that hypoxic conditions occur during luteinization, thus favoring the formation of L-lactate within the follicle. Despite being a product of anaerobic respiration, L-lactate has been shown to act as a signaling molecule affecting gene expression in neuronal cells. During the present study, we tested the hypothesis that L-lactate may influence differentiation of follicular granulosa cells (GC).

**Methods:**

In a bovine granulosa cell culture model effects of L- and D-lactate, of increased glucose concentrations and of the lactate transport inhibitor UK5099 were analyzed. Steroid hormone production was analyzed by RIA and the abundance of key transcripts was determined by quantitative real-time RT-PCR.

**Results:**

L-lactate decreased the production of estradiol and significantly affected selected genes of the folliculo-luteal transition as well as genes of the lactate metabolism. *CYP19A1*, *FSHR*, *LHCGR* were down-regulated, whereas *RGS2*, *VNN2*, *PTX3*, *LDHA* and lactate transporters were up-regulated. These effects could be partly or completely reversed by pre-treatment of the cells with UK5099. The non-metabolized enantiomer D-lactate had even more pronounced effects on gene expression, whereas increased glucose concentrations did not affect transcript abundance.

**Conclusions:**

In summary, our data suggest that L-lactate specifically alters physiological and molecular characteristics of GC. These effects critically depend on L-lactate uptake, but are not triggered by increased energy supply. Further, we could show that L-lactate has a positive feedback on the lactate metabolism. Therefore, we hypothesize that L-lactate acts as a signaling molecule in bovine and possibly other monovular species supporting differentiation during the folliculo-luteal transformation.

**Electronic supplementary material:**

The online version of this article (10.1186/s12958-018-0332-3) contains supplementary material, which is available to authorized users.

## Background

The pre-ovulatory LH surge induces massive changes within the follicle, including morphological, physiological and molecular alterations [1, 2]. The reorganization of the follicle is accompanied by a tremendous shift of the gene expression profile particularly in cells of the granulosa layer [3, 4]. The aromatase-encoding gene *CYP19A1*, and the gonadotropin receptors, *FSHR* and *LHCGR*, are significantly down-regulated immediately after the pre-ovulatory LH surge in vivo [5, 6]. In contrast, *RGS2*, encoding the regulator of G protein signaling 2, and the inflammatory-related genes *VNN2*, encoding the vascular non-inflammatory molecule 2, and *PTX3*, encoding pentraxin 3, were highly up-regulated [4, 7, 8]. It was hypothesized that hypoxic conditions occur during folliculogenesis and support ovulation [9, 10]. However, it is still unclear whether hypoxia is an essential signal during the folliculo-luteal transition [11]. As for granulosa cells (GC) it was proposed that pericellular hypoxia present under high cell density conditions may induce differentiation [12]. Likewise, we showed that high cell density induces specific changes of the gene expression and steroid hormone profiles in cultured GC mimicking an early post-LH status of GC differentiation [13]. Furthermore, applying a whole-genome approach we could demonstrate in GC cultured at high plating density, that genes connected to hypoxia are affected [14]. In particular *LDHA* transcripts encoding L-lactate dehydrogenase were remarkably up-regulated. It was demonstrated also by others that *LDHA* expression is regulated by hypoxia [15]. In addition, a binding site for HIF1/2α could be identified in the *LDHA* promotor explaining the hypoxia-related expression [16, 17]. On the other hand Lee et al. [18] proposed a HIF-independent mechanism of lactate accumulation under hypoxic conditions. Hence, L-lactate might play a role during the folliculo-luteal transition. Reports from different species demonstrated higher L-lactate concentrations within the follicular fluid than in the respective serum, ranging from 6 mM in human up to 27 mM in rats [19–21]. Generated even during adequate oxygen provision L-lactate might represent an important regulator of metabolism [22]. A study in L6 cells revealed that L-lactate may be involved in the delivery of oxidative and gluconeogenic substrates thus leading to the cell-cell and intracellular lactate shuttle hypothesis [23, 24]. In this context L-lactate is also described as a metabolic signal. L-lactate affects its own metabolism by stimulating the expression of the lactate transporter MCT1 in rat muscle cells [25]. In mouse granulosa cells MCTs were identified regulating the transport of L-lactate within the female reproductive tract [26]. Moreover, in neuronal cells L-lactate affects the expression of genes linked to neuronal plasticity during establishment of the long-term memory [27, 28]. In this study we therefore tested the hypothesis that L-lactate is a signaling molecule in the bovine follicle. To that end, we analyzed the effects of L-lactate in a serum-free estradiol (E2)-producing GC culture model [13, 29, 30] on specific morphological, physiological and molecular parameters.

## Methods

### Tissue collection and cell culture

Bovine ovaries were obtained from a local abattoir and transported in cold 1× PBS containing penicillin (100 IU), streptomycin (0.1 mg/ml) and amphotericin (0.5 μg/μl). By aspirating small to medium sized follicle (< 6 mm) with a syringe and 18 G needle a nearly pure population of granulosa cells was recovered [5]. GC were collected in 1× PBS (with antibiotics) and pooled from 30 to 50 ovaries with 15 to 30 follicles per ovary. Accordingly, samples from at least 15 different cows with non-defined cyclicity status were included in each preparation. To determine the amount of living cells GC were counted in a hemocytometer by trypan blue exclusion method and cryo-preserved in freezing media (fetal calf serum containing 10% DMSO; Roth, Karlsruhe, Germany). The cryo-preserving procedure did not alter general characteristics of GC in culture as we could detect equal levels of marker gene expression as compared with cultures from freshly isolated GC (Additional file 1: Figure S1). All experiments were performed in technical and biological replicates (at least 3) using different cell preparations. Shortly before the onset of cell culture 24-well plates were coated with collagen R (0.02%; Serva, Heidelberg, Germany) to improve the cell attachment [13]. Frozen GC were immediately separated from the freezing media by centrifugation (500 x *g* for 3 min) and re-suspended in culture media with supplements as described previously [13]. Briefly, cells of a total cell number of 1.0 × 10^5^ cells/well were cultured under serum-free conditions in α-MEM containing L-Glutamin (2 mM), sodium bicarbonate (0.084%), BSA (0.1%), HEPES (20 mM), sodium selenite (4 ng/ml), transferrin (5 μg/ml), insulin (10 ng/ml), non-essential amino acids (1 mM), penicillin (100 IU/ml) and streptomycin (0.1 mg/ml), FSH (20 ng/ml; Sigma Aldrich, Steinheim, Germany), R^3^ IGF-1 (50 ng/ml; Sigma Aldrich), and androstenedione (2 μM; Sigma Aldrich). The cells were additionally treated over the complete culture period with sodium L-lactate (7.5 mM – 30 mM), D-lactate (30 mM, both Sigma-Aldrich), or sodium chloride as vehicle control (7.5 mM – 30 mM; Sigma Aldrich). For inhibitory studies cells were initially pre-treated with inhibitor UK5099 (50 μM; Sigma Aldrich) for 48 h and subsequently treated with L-lactate or the vehicle control. The L-lactate transporters were inhibited with UK5099 (50 μM; Sigma Aldrich). All reagents were purchased from Merck Millipore (Berlin, Germany) if not stated otherwise. GC were maintained for 8 days at 37 °C and 5% CO_2_ to allow the cells to completely recover from plating stress and to resume E2 production [31]. Two thirds of the spent culture medium was replaced every other day. Also in previous studies it has been repeatedly shown that GC obtained from small follicles (< 6 mm) require several days in vitro to develop an estradiol-active status that mimics the in vivo GC state shortly before the LH surge [13, 32].

### Measurements of cell viability and hormone production

The cell viability test was performed using the CellTiter 96 AQueous One Solution Cell Proliferation Assay (Promega, Mannheim, Germany). The one compound solution contains a MTS tetrazolium salt which is converted into a colored formazan product by NAD(P)H that is only present in viable cells. Therefore, the quantitative detection of the formazan product by absorbance measurement represents the number of viable cells in culture. The assay was performed according to the manufacturer’s protocol. Therefore, cells were cultured in collagen R coated 96-well plates. The absorbance was measured in a 96-well plate reader (FLUOstar Omega, BMG Labtech, Ortenberg, Germany) at a wavelength of 490 nm. Progesterone (P4) and E2 concentrations were measured using competitive ^3^H–radioimmunoassay (RIA) as described previously [33]. For P4 the tracer [1,2,6,7-3H(N)]progesterone was purchased from PerkinElmer (Boston, USA) and the E2 tracer [2,4,6,7-3H]estradiol-17β was purchased from GE Healthcare (Freiburg, Germany). Radioactivity measurement was performed in a liquid scintillation counter (LSC) with an integrated RIA-calculation program (TriCarb 2900 TR, PerkinElmer) and the intra- and interassay coefficients of variation (CV) for P4 were specified as 7.6% and 9.8%, whereas for E2 intra- and interassay CV were 6.9% and 9.9%, respectively. For analysis the cell culture media was diluted 1:40 in RIA-buffer for P4 measurement and was used undiluted for E2 and measured in duplicates.

### RNA preparation, cDNA synthesis and quantitative Real-Time PCR

Total RNA isolation was performed with the NucleoSpin RNA Kit (Macherey-Nagel, Düren, Germany) following the protocol of the manufacturer and RNA concentration was measured using a NanoDrop 1000 Spectrophotometer (Thermo Scientific, Bonn, Germany). Subsequently, cDNA was synthesized with SensiFAST cDNA Synthesis Kit (Bioline, Luckenwalde, Germany) from 250 ng RNA. Quantitative Real-Time PCR for gene expression analysis was accomplished with SensiFast SYBR No-ROX (Bioline) and gene-specific primers (listed in Table 1). Complementary DNA samples were amplified in duplicate from 0.2 and 0.4 μl in a total volume of 12 μl. The PCR was performed and quantified in a LightCycler 96 instrument (Roche, Mannheim, Germany) with following cycle conditions: pre-incubation at 95 °C for 5 min, 40 amplification cycles of denaturation at 95 °C for 20 s, annealing at 60 °C for 15 s, extension at 72 °C for 15 s, and a single-point fluorescence acquisition for 10 s. Analysis of the melting point was done immediately afterwards to ensure amplification of correct products. For the same reason the length of PCR products was checked by agarose gel electrophoresis (3%, ethidium bromide stained). Cloned and sequenced PCR products were used as external standards for absolute quantification. Of these, fresh dilutions were prepared from five different concentrations of standards (5 × 10^− 12^-5 × 10^− 16^ g DNA/reaction) and co-amplified in each run. The absolute quantified transcript abundance was normalized to the reference gene *RPLP0*, showing stable expression values throughout the different treatments.

**Table 1:** Gene specific primers used in quantitative Real-Time PCR

Gene	dir	Sequence	Size (bp)	Accession No.
*RPLP0*	for	TGGTTACCCAACCGTCGCATCTGTA	142	NM_001012682
	rev	CACAAAGGCAGATGGATCAGCCAAG		
*CYP19A1*	for	GCTTTTGGAAGTGCTGAACCCAAGG	172	NM_174305
	rev	GGGCCCAATTCCCAGAAAGTAGCTG		
*FSHR*	for	TCACCAAGCTTCGAGTCATCCCAAA	189	NM_174061
	rev	TCTGGAAGGCATCAGGGTCGATGTA		
*LHCGR*	for	GCATCCACAAGCTTCCAGATGTTACGA	205	NM_174381
	rev	GGGAAATCAGCGTTGTCCCATTGA		
*RGS2*	for	AAGCCCAGCTGTGGTCAGAAGCATT	127	NM_001075596
	rev	TCTTCACAGGCCAGCCAGAATTCAA		
*VNN2*	for	TCCCACAGCTTGGATGAACGTTTTG	267	NM_001163920
	rev	TAGGCACTCCAATTCATGGCTGGTG		
*PTX3*	for	TTTGTGCGCTCTGGTCTGCAGTGT	164	NM_001076259
	rev	CATGGTGAAGAGCTTGTCCCACTCG		
*LDHA*	for	GGGTTGGTGCTGTTGGCATGGCCT	232	NM_174099.2
	rev	TCTCCCTCTTGCTGACGTGCCCCA		
*SLC16A1*	for	GGTGGAGGTCCTATCAGCAGTGTCCT	236	NM_001037319.1
	rev	AGTCCATTTGCCAGCGGTCGTCTC		
*SLC16A7*	for	ACCCAGTGCCGGAGACCAGCAGTT	182	NM_001076336.2
	rev	GGATGTGGTGGTGGGGTGCCTCCT		

dir, direction; for, forward primer; rev, reverse primer

### Statistics

Each experiment was done with at least three biological replicates (different GC preparations) and three technical replicates (different culture wells) each. SigmaPlot 12.0 Statistical Analysis System (Jandel Scientific, San Rafael, CA, USA) was used for statistical analysis. Statistical analysis of hormone and qPCR measurement was carried out with untransformed data. Comparison of two different treatments (e.g. L-lactate vs. vehicle control) was performed with the student *t*-test or Mann-Whitney Test, when the requirements of the t-test were not fulfilled. Multiple comparisons were done with one-way analysis of variance (ANOVA, Holm-Sidak method). Limits of statistical significance were set at *P* < 0.05. For graphical presentation the ordinate was scaled in log2 (except for glucose experiment), which distributes the effects of up- and down-regulation more uniformly.

## Results

### Effects of L- and D-lactate on hormone production and gene expression

**Fig. 1:**
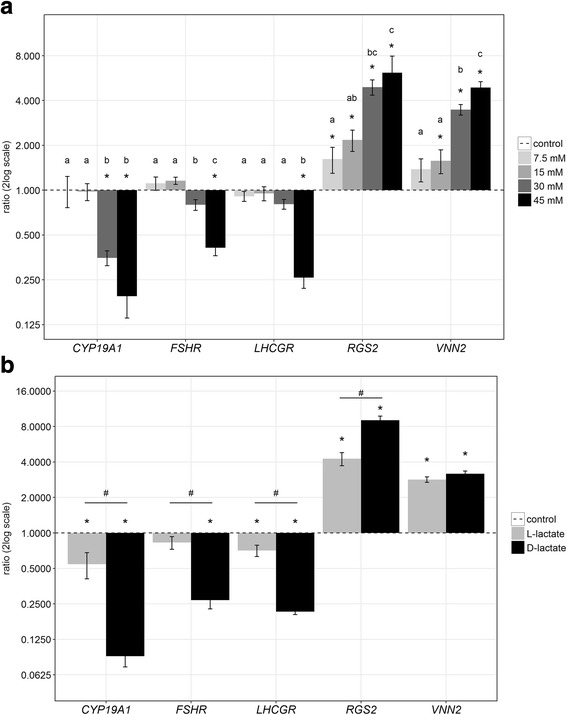
Effects of different L-lactate concentrations and of the steric enantiomer D-lactate on cultured bovine GC. (**a**) Increasing L-lactate concentrations progressively decreased or increased the abundance of selected marker gene transcripts. Transcript abundance was normalized to *RPLP0* as a reference gene. The values (means ± SEM) are shown as ratio relatively to the vehicle control (dashed line). For statistical testing of the L-lactate effect the *RPLP0*-normalized values were compared using the student *t*-test or Mann-Whitney test, *n* = 3, *P < 0.05. To test for effects of increasing L-lactate concentration an ANOVA was performed and letters indicate significant differences between treatment groups, *n* = 3, *P* < 0.05. (**b**) The supplementation with D-lactate (30 mM) elicited more prominent effects on gene expression than L-lactate (30 mM) compared to the vehicle control (dashed line). The student’s *t*-test or Mann-Whitney test was used for analyzing the L- and D-lactate effects compared to the respective control (*) and between L- and D-lactate treatment (#), *n* = 3, *P* < 0.05.

Initially, effects of different L-lactate concentrations on cultured GC were analyzed. Previous studies reported effective concentrations of 20 mM or 30 mM in different cell types [27, 34, 35]. In various species L-lactate concentrations in the antral fluid of growing follicles could be detected between 6 and 27 mM [19, 21]. Therefore, we treated the GC with L-lactate concentrations ranging from 7.5 mM to 45 mM and corresponding vehicle controls (NaCl). As highly sensitive parameters we determined the abundance of several marker transcripts, which are highly regulated in the GC layer during the folliculo-luteal transition [4]. The treatment with 30 mM and 45 mM L-lactate resulted in considerable changes of these molecular parameters compared to the control (Fig. 1a). We could identify a dose-dependent down-regulation of *CYP19A1, FSHR* and *LHCGR*. *RGS2* and *VNN2* transcripts revealed a dose-dependent up-regulation. Whereas a significant up-regulation of *RGS2* expression was already achieved with 7.5 mM L-lactate, a significant down-regulation of *CYP19A1* expression was observed with 30 mM L-lactate. We therefore considered 30 mM L-lactate as an effective concentration during the ongoing study of in vitro cultured GC.

In studies by others D-lactate, a non-metabolized and iso-osmotic enantiomer of L-lactate, was used as a control [27, 36]. However, in GC the supplementation with D-lactate led to even more pronounced and specific changes of several marker genes (Fig. 1b). The down-regulation of *CYP19A1*, *FSHR* and *LHCGR* by D-lactate revealed to be more prominent than with L-lactate. The same effect could be observed for the up-regulation of *RGS2* and *VNN2*. Accordingly, D-lactate could not be used as an appropriate control.

**Fig. 2:**
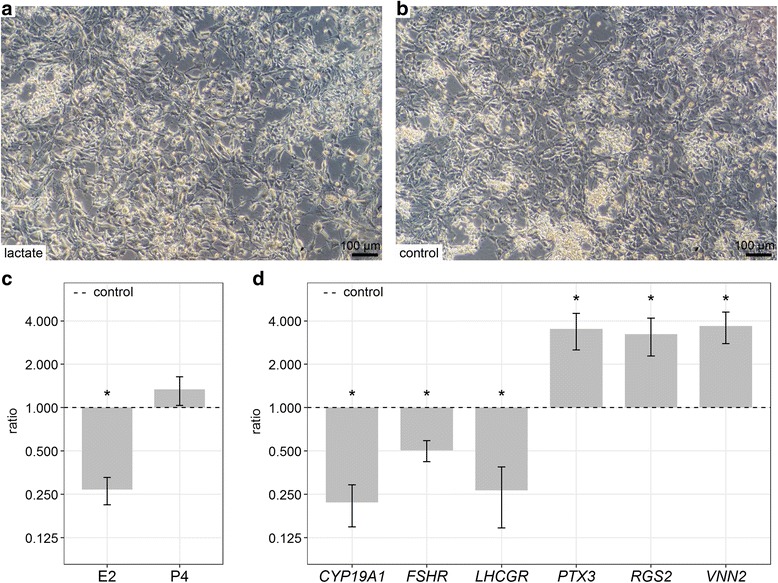
Effects of L-lactate on the morphology, hormone concentration and gene expression. GC were cultured with L-lactate (30 mM) supplementation compared to the corresponding vehicle control in a standardized serum-free culture system for 8 days. (**a**-**b**) Cultured cells displayed a typical GC-like morphology and tended to form clusters. (**c**) Hormone profiles of L-lactate treated cells indicated relatively to the control (dashed line). The concentration of E2 (in ng/ml) was significantly decreased relative to the vehicle control (dashed line). (**d**) The expression of selected marker genes was specifically regulated in L-lactate treated cells as compared to the vehicle control (dashed line). The transcript abundance was normalized to the reference gene *RPLP0*. Means and SEM are shown of *n* = 3 replicates, *P < 0.05, student *t*-test or Mann-Whitney test.

Further on we analyzed whether L-lactate or the vehicle control (NaCl) compromised the viability of cultured GC. Although the viability tended to be slightly affected in L-lactate treated cells, these effects were not statistically significant (Table 2). As a paradigm for differentiation-specific changes of cultured GC the morphology, hormone concentrations as well as the expression of GC-specific marker genes were analyzed in L-lactate-treated GC. As shown in Fig. 2a and b the morphology of cultured GC was not considerably altered. GC displayed the typical fibroblast-like phenotype with occasional cluster formation under both experimental conditions. In contrast, the estradiol production was clearly affected showing considerably lower concentration (Fig. 2c). Additionally, the abundance of several selected marker transcripts revealed a significant regulation upon L-lactate treatment (Fig. 2d). The abundance of *CYP19A1* transcripts encoding the key enzyme of estradiol synthesis, aromatase P450, was significantly lower in L-lactate treated GC than in the controls (dashed line). This is also reflected by the observed decrease of E2 production. Similarly, the amounts of *FSHR* and *LHCGR* transcripts were significantly down-regulated. In contrast, *RGS2*, *VNN2* and *PTX3* transcripts were considerably up-regulated by L-lactate.

**Table 2:** Effects of lactate and NaCl control on the viability of cultured bovine GC

treatment	untreated	lactate	NaCl
OD	0.997	0.640	1.291
SEM	0.245	0.177	0.165

The cells were cultured for 8 days under serum-free, E2-active conditions, with L-lactate or the vehicle control NaCl (both 30 mM) supplementation. The mean OD (optical density) of *n* = 4 replicates is shown with SEM (standard error of means), no significant differences between treatment groups were observed, *P* = 0.108 (ANOVA)

**Fig. 3:**
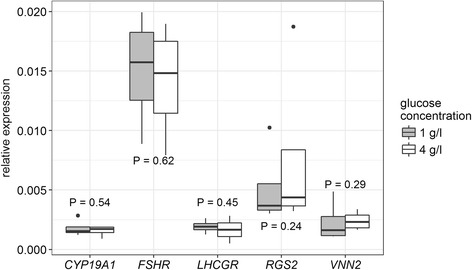
Effects of different glucose concentration in media. GC were cultured in basal media with 1 g/l glucose (grey) or increased concentration of 4 g/l glucose (black). No change in the expression of selected genes could be detected. Transcript abundance was normalized to reference gene *RPLP0.* Box plots with medians of *n* = 4 replicates are shown; dots indicate outliers, student *t*-test or Mann-Whitney test with no statistical significance.

In addition to these long-term effects of L-lactate, we were interested whether a short-term treatment is sufficient to induce these massive physiological and molecular changes. Thus, the cultured GC were treated with 30 mM L-lactate during the final 24 h of the culture period. Under these conditions no significant alterations of marker transcripts as well as of hormone concentrations could be observed (Table 3). This indicates that L-lactate has only long-term effects on GC differentiation. Additionally, because increased L-lactate concentrations might be used as an additional source of energy by the cultured GC, we analyzed whether an increased energy supply suggestively resulting in higher metabolic activity of the cells, could be responsible for the effects of L-lactate. Therefore we increased the glucose concentration of the basal media from 1 g/l up to 4 g/l. The 4-fold increase of glucose concentration however, did not lead to any changes in the expression of the selected marker genes (Fig. 3).

**Table 3:** Effects of short-term lactate treatment on gene expression of selected marker genes and steroid hormone production

*Gene* / hormone	*CYP19A1*	*FSHR*	*LHCGR*	*RGS2*	*VNN2*	E2	P4
Control	3.12 (± 0.31)	34.70 (± 4.41)	3.29 (± 0.46)	5.56 (± 0.29)	6.26 (± 0.39)	23.72 (± 1.57)	824.61 (± 84.99)
L-lactate	3.78 (± 0.85)	49.10 (± 8.33)	3.18 (± 0.94)	5.78 (± 0.32)	7.39 (± 1.11)	32.31 (± 5.26)	905.01 (± 47.03)
P	0.50	0.20	0.92	0.63	0.39	0.19	0.45

GC were stimulated with 30 mM L-lactate or the vehicle control NaCl 24 h prior to the end of the culture period of 8 days. Transcript abundance (×10^−3^, normalized to the reference gene *RPLP0*) and hormone concentrations (in ng/ml) are shown as means (± SEM) of *n* = 3 replicates, *P*-values were calculated using the student’s *t*-test or Mann-Whitney Test

### Effects of specific inhibition of L-lactate transporters

**Fig. 4:**
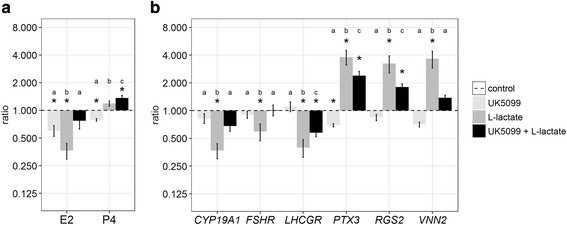
Effects of L-lactate transport inhibitors on L-lactate induced changes of hormone and gene expression profile. GC were cultured as described before and additionally pre-treated for two days with the inhibitor for L-lactate transporters, UK5099 (50 μM). (**a**) Inhibition of L-lactate transporters (black bars) resulted in the reversal of the L-lactate effect (dark grey bars) on E2 production. (**b**) The gene expression levels of selected transcripts nearly regained control levels (dashed line) after inhibition of L-lactate transporters, whereas the inhibitor alone showed nearly no effects (light grey bars). Transcript abundance was normalized to the reference gene *RPLP0*. Means and SEM of the hormone concentration and gene expression are shown of *n* = 4 replicates. The student’s *t*-test or Mann-Whitney test was used for analyzing the effects compared to the vehicle control (*) and between different treatments the ANOVA testing was used (indicated by letters), *n* = 4, *P* < 0.05.

As a first approach to elucidate molecular mechanisms that are involved in the observed effects of L-lactate we pre-treated GC with specific inhibitor of lactate transporters (MCTs). Inhibition of MCTs with UK5099 could partly reverse the L-lactate effect (Figs. 4a and b). Negative effects of L-lactate on E2 production could be abolished (Fig. 4a). The inhibition of MCTs also resulted in a recovery of the transcript abundance levels of *CYP19A1* and *FSHR* (Fig. 4b). Also in case of *PTX3*, *RGS2* and *VNN2* the L-lactate-induced increment was reduced, although the transcript abundance was still significantly higher than in the control, except for *VNN2*. The treatment with the inhibitor alone resulted in no obvious changes compared to the vehicle control.

### Effects of L-lactate and specific MCT inhibitor on genes related to L-lactate metabolism

**Fig. 5:**
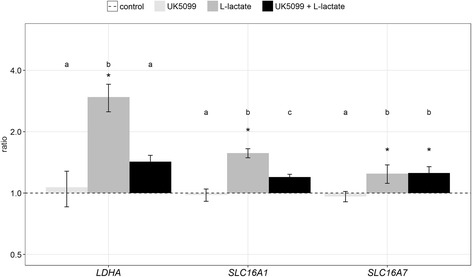
Effect of L-lactate and specific inhibitor of L-lactate transport on genes involved in L-lactate metabolism. The abundance of *LDHA* and *SLC16A1* transcripts was up-regulated in L-lactate treated GC (grey bars) compared to the control (dashed line; *). When the cells were pre-incubated with the inhibitors UK5099 the L-lactate induced changes of expression levels were largely abolished. No effect was observed with the inhibitor alone (light grey bars). Transcript abundance was normalized to *RPLP0* as a reference gene. Means and SEM are shown of *n* = 4 replicates, *P < 0.05, student’s *t*-test or Mann-Whitney test was uses to test treatment effects compared to the vehicle control (*) and between different treatments the ANOVA was used (indicated by letters).

It was recently reported that L-lactate can influence genes related to the L-lactate metabolism resulting in a positive feedback mechanism [25]. Therefore, we were interested whether the genes encoding L-lactate dehydrogenase, *LDHA*, or L-lactate transporters, *SLC16A1* and *SLC16A7*, were affected. The *LDHA* transcript abundance was strongly increased upon L-lactate treatment (Fig. 5), but also the L-lactate transporters *SLC16A1* and *SLC16A7* showed a significant up-regulation. After pre-treatment with UK5099, the L-lactate effects on the expression of *LDHA* and *SLC16A1* could be nearly reversed.

## Discussion

Initial experiments with different lactate concentrations revealed significant effects on *RGS2* expression with 7.5 mM and on *CYP19A1* or *FSHR* expression with 30 mM. *LHCGR* was significantly affected only with 45 mM. However, this might be due to the generally observed greater variance of *LHCGR*, of which the very low abundance levels were close to the detection limit. Nonetheless, based on these data, an L-lactate concentration of 30 mM was used as an effective concentration throughout the following experiments. Interestingly, the non-metabolized enantiomer D-lactate elicited more prominent effects on gene expression than L-lactate. An effect of D-lactate was also described by Latham et al. [35] and Wagner et al. [37], analyzing HDAC activity. However, it must be considered that the concentrations of D-lactate in mammalian species has been shown to be three orders of magnitude lower than those of L-lactate [38], thus raising the question whether D-lactate plays any important role after all. But nonetheless, our data on increased effects of the non-metabolized D-lactate support our hypothesis that L-lactate can act as a signaling molecule apart from being metabolized. Moreover, it is reasonable that the enhanced effects of D-lactate are due to the non-metabolized status in mammalian cells. At this point we cannot exclude the possibility that a part of L-lactate is metabolized, which in turn leads to a reduction of L-lactate concentration and hence results in minor effects than the non-metabolized D-lactate. This leads to the suggestion that chirality might not be the primary parameter for signaling efficiency. This hypothesis has to be further taken into account in subsequent studies.

Interestingly, our results of different glucose concentration in GC media revealed no alterations in the gene expression. This indicates that an increased energy supply and thus higher metabolic activity alone is not sufficient to induce differentiation of bovine GCs in culture, which was shown in a previous work in sheep GC [39].

In follicular fluids of rats L-lactate concentrations of 27 mM had been detected at the time of the expected LH surge, when the serum concentration of L-lactate remained considerably low at 5 mM [21]. Studies in humans showed concentrations of 6.2 mM L-lactate at the time of oocyte recovery from women undergoing IVF [19]. In the bovine different values have been reported ranging from 5 mM up to 14.4 mM depending on the size or stage of the follicle [20, 40, 41]. Until now, effects of L-lactate on the cell physiology and gene expression have been only studied in rat L6 cells, human mesenchymal stem cells, human HCT116 cells or mouse neurons, where effective concentrations ranged from 10 to 30 mM lactate [27, 34, 35, 42]. To our knowledge the present study is the first one that aims at clarifying the effects of L-lactate on GC differentiation in any species.

Short-term treatment of cultured bovine GC at the end of the culture period did not lead to considerable alterations. Neither hormone production nor gene expression profiles were significantly affected. Interestingly, this observation is in line with a conclusion by others that short-term exposure to L-lactate does not affect gene expression, but may already have some effects on the cell metabolism [23]. Furthermore, it was suggested that effects on gene expression potentially require long-term exposure to L-lactate [23]. This is clearly supported by our data showing that only long-term treatment could induce significant changes of physiological and molecular parameters in cultured GC, although the typical GC-like morphology and formation of cell clusters [13, 30, 32] was not affected by L-lactate. Estradiol production clearly decreased whereas progesterone production was nearly unaffected compared to the controls. Estradiol down-regulation can be easily attributed to the remarkable down-regulation of the aromatase coding gene *CYP19A1*. Besides *CYP19A1* we also analyzed additional marker genes of an early LH-dependent differentiation, which have been shown to be highly regulated following the pre-ovulatory LH-surge in vivo [4, 5, 7]. In particular, *FSHR* and *LHCGR* were found down-regulated and *RGS2*, *VNN2* and *PTX3* clearly up-regulated upon L-lactate treatment. These data together with the alterations of the hormone production suggest that L-lactate can induce a stage of differentiation in cultured GC that is similar to the early post-LH stage in vivo. Previous studies identified increased L-lactate concentrations after hCG stimulation in human GC culture or in the antral fluid of follicles in macaques thus supporting our conclusion [43, 44]. L-lactate is well known as a product of anaerobic respiration, but may also play a role as regulatory molecule even during adequate oxygen provision [22, 24]. Interestingly, it was described in several studies that L-lactate can act as a signaling molecule in fibroblasts [45], macrophages [46] and even neuronal cells [27, 28]. Yang and colleagues showed that in neurons L-lactate affected the expression of genes involved in the establishment of long-term memory [27]. In another recent study L-lactate exhibited neuroprotective properties against excitotoxicity by coordinating specific cellular pathways [47]. The results of our study indicate for the first time a specific role of L-lactate as a signaling molecule in bovine GC.

As a next approach we tested if L-lactate uptake is an essential prerequisite to induce the observed effects. The inhibition of L-lactate uptake was accomplished with UK5099, a potent MCT inhibitor of L-lactate transport [27]. This inhibitor is characterized to target the group of MCT 1–4 that are responsible for lactate as well as pyruvate transport. Accordingly, the transport of L-lactate into the cells should be completely inhibited by UK5099. Our results showed that the estradiol concentrations as well as the expression of selected marker genes nearly regained control levels after inhibiting the MCTs. This clearly suggests that the observed effects are vitally dependent upon L-lactate uptake into GC. It has been shown in neurons, that transport of L-lactate into the cells and K_ATP_ channels [48] or NMDA receptor activation [27] is necessary to promote the observed changes. In contrast to our data demonstrating strong effects of the non-metabolized D-lactate, the conversion of L-lactate to pyruvate is a crucial prerequisite in both cases. Whereas the rise of ATP production is essential for K_ATP_ channel actions, the increase in the NADH/NAD ratio leads to the modulation of NMDA receptor activity [28]. Here we can only speculate on mechanisms occurring in bovine GC. But in human ovaries functional ATP-sensitive potassium channels could be identified and moreover it was shown that blocking of these ion channels negatively influences progesterone production [49]. In ovarian tissue and granulosa cells expression of specific subunits of the NMDA receptor, the subunits 1 and 2B, had been identified [50] thus supporting the view that this receptor might be involved in these cells as well. Expression of NMDAR subunits, especially of *GRIN2D* and *GRINA* has been found in vivo in GC (see database GranulosaIMAGE [51]) as well as in vitro (own GC culture microarray dataset [14]). Whether these receptor subunits can actually form functional receptors to be modulated by L-lactate in bovine GC has to be investigated in further studies. However, our data clearly show that L-lactate uptake is mandatory for most of the observed effects thus excluding the possibility that a G-protein coupled receptor for lactate, coded by *HCAR1*, might be necessary to carry the L-lactate signal across the cell membrane [52]. In addition, *HCAR1* transcripts were not detectable in our GC culture system (data not shown) thus indicating that *HCAR1* is not expressed in cultured GC. This is further supported by in vivo data demonstrating that *HCAR1* expression could not be detected in GC [51]. The mode of action of L-lactate within bovine GC, however, is still an open question and has to be further elucidated.

At present, we cannot completely exclude the possibility that endogenous L-lactate production by the cultured cells might contribute to the observed effects. However, in an initial study without any lactate treatment we measured lactate concentrations in the media of approximately 7 mM (not shown), which is far from the effective concentrations used during the presented study. Therefore, it is reasonable to assume that endogenously produced lactate did not significantly interfere with our experimental settings.

The present results demonstrate that L-lactate has positive effects on the expression of *LDHA*, the enzyme regulating the L-lactate-pyruvate-ratio as well as on the expression of its transporters, *SLC16A1* and *SLC16A7*. This is in accordance with earlier observations by others that L-lactate directly stimulates the expression of the MCT1 protein (encoded by *SLC16A1*) in L6 cells [34]. Another study showed that endurance training, leading to a higher L-lactate production, increased MCT1 expression in rats and humans [23]. This suggests that also in bovine GC L-lactate can stimulate its own metabolism and transport, thus being part of a positive feedback loop. Supportively, in skeletal muscle cells it was shown that MCT1 expression and L-lactate uptake is dependent on the activation of PKA and PKC signaling [53]. This is an interesting hypothesis, having in mind that the PKA signaling is one pathway involved in inducing luteinization of GC [54].

## Conclusions

The presented data on hormone production and expression of marker genes clearly suggest that L-lactate treatment of cultured bovine GC results in a differentiation similar to early LH-induced alterations in vivo. Furthermore, the data suggest that L-lactate is involved in a positive feedback mechanism regulating its own transport and metabolism. The accumulation of L-lactate within the follicular fluid [19–21, 40] suggests that L-lactate also plays a role in vivo as a differentiation factor during late folliculogenesis and the folliculo-luteal transition in monovular species. In subsequent studies this hypothesis of the impact of lactate on GC differentiation has to be proven in in vivo models as well.

## Additional file

Additional file 1: **Figure S1.** Comparison of the expression of marker genes in GC cultured immediately after isolation vs. GC cultured after cryo-preservation. No difference between GC cultured either directly after isolation or after cryo-preservation could be observed. Transcript abundance is shown as absolute expression (copy number per μg RNA) of *n* = 3, student’s *t*-test revealed no significant differences. (TIFF 226 kb)

## Abbreviations

BSA: Bovine serum albumin
CV: Coefficient of variation
*CYP19A1*: Aromatase
DMSO: Dimethyl sulfoxide
E2: Estradiol
FSH: Follicle stimulating hormone
*FSHR*: FSH receptor
GC: Granulosa cells
*GRIN2D*: Glutamate ionotropic receptor NMDA type subunit 2D
*GRINA*: Glutamate ionotropic receptor NMDA type subunit associated protein 1
*HCAR1*: Hydroxycarboxylic acid receptor 1
hCG: Human choriogonadotropin
HDAC: Histone deacetylase
HIF1/2α: hypoxia-inducible factors 1/2 alpha
IGF-1: Insulin-like growth factor 1
*LDHA*: Lactate dehydrogenase
LH: Luteinizing hormone
*LHCGR*: LH/choriogonadotropin receptor
MCT: Monocarboxylate transporter
MEM: Minimal essential media
NMDAR: N-methyl-D-aspartate receptor
P4: Progesterone
PBS: Phosphate buffered saline
PKA: Protein kinase A
PKC: Protein kinase C
*PTX3*: Pentraxin 3
qPCR: quantitative PCR
RIA: Radioimmunoassay
*RGS2*: Regulator of G protein signaling 2
*SLC16A1*: Solute carrier family 16 member 1
*SLC16A7*: Solute carrier family 16 member 7
UK5099: 2-Cyano-3-(1-phenyl-1H-indol-3-yl)-2-propenoic acid
*VNN2*: Vanin 2
