# Supplementary figures and images for: Lactate promotes specific differentiation in bovine granulosa cells depending on lactate uptake thus mimicking an early post-LH stage
Source: Reprod Biol Endocrinol. 2018 Feb 20;16:15. doi: 10.1186/s12958-018-0332-3 (PMC5819637; doi:10.1186/s12958-018-0332-3)

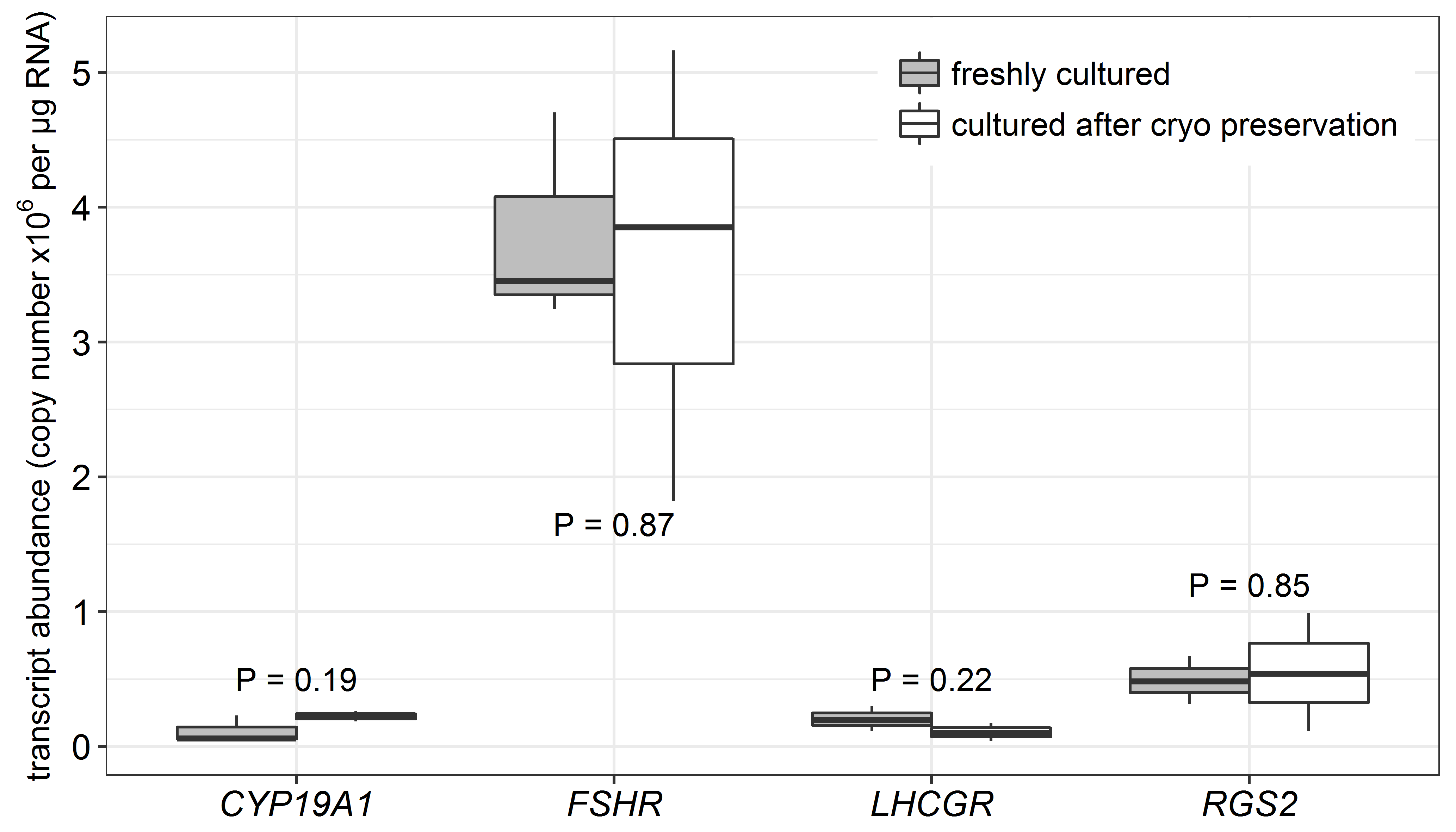

Supplement: Supplementary file 1 — Figure S1. Comparison of the expression of marker genes in GC cultured immediately after isolation vs. GC cultured after cryo-preservation. No difference between GC cultured either directly after isolation or after cryo-preservation could be observed. Transcript abundance is shown as absolute expression (copy number per μg RNA) of n = 3, student’s t-test revealed no significant differences. (TIFF 226 kb) [file 12958_2018_332_MOESM1_ESM.tif]
